# Memoirs of an alcoholic

**Published:** 2010

**Authors:** Sanju George

**Affiliations:** Consultant and Senior Research Fellow in Addiction Psychiatry, Birmingham and Solihull Mental Health NHS Foundation Trust, England, UK. E-mail: sanju.george@bsmhft.nhs.uk

Don't ask me how it started
It was cool when it started
Cool it isn't anymore
Don't ask me where it all went wrong
Don't ask me if I'm responsible
I want to but I can't stop.

It makes me happy, not forever
It helps me forget, not forever
I still crave for that little solace
It hurts to say but I'm in love
Love a drink, love beyond reason
Drink until death cometh knocking.

I hate the morning-after headaches
I hate the nagging wife, I hate the guilt
But I have a drink and it's all worth it
I've tried hard but I failed
I've tried pills and implants
But see the boozer and I falter

Times I wish it would all end
I'm not guilty for I'm not in control
Am I a lesser man for that?
In pain and misery I drown
For answers I long, In vain I seek
May be it was my dad, may be it was my teacher,
Or is it because I'm sad and lonely?

Would anyone care if I was gone?
Would anyone hurt?
Lay me to rest
A bottle of gin by my side
For friendships are forever
Through times rough and smooth
She was by my side

